# COVID-19: using the crisis as an opportunity to strengthen Primary Health Care

**DOI:** 10.1017/S1463423621000748

**Published:** 2021-11-22

**Authors:** Jan De Maeseneer

**Affiliations:** Family Physician. Head WHO Collaborating Centre on Family Medicine and PHC – Ghent University. E-mail: jan.demaeseneer@ugent.be

The COVID-19 pandemic had impact not only on the health system but also on the whole societal fabric. Hence, it has correctly been labeled by Richard Horton as a ‘syndemic’ (Horton, 2020). Syndemics are characterized by biological and social interactions between conditions and states, interactions that increase a person’s susceptibility to harm or worsen their health outcomes. This is one of the reasons why the performance and strength of Primary Health Care (PHC) is highlighted in all analyses as one of the most important determinants of outcome, both at the level of individuals and at population level. This relates to one of the most important conclusions of the WHO report ‘*Closing the gap in a generation*’, produced by the Commission on Social Determinants of Health in 2008, under the leadership of Sir Michael Marmot: ‘*Health-care systems have better health outcomes when built on Primary Health Care (PHC) – that is, both the PHC model that emphasizes locally appropriate action across the range of social determinants, where prevention and promotion are in balance with investment in curative interventions, and an emphasis on the primary level of care with adequate referral to higher levels of care’* (WHO, 2008 p.12).

Several reports echo the important contribution of PHC in helping health systems adapt during the COVID-19 pandemic. In January 2021, an Organisation for Economic Co-operation and Development (OECD) report on ‘*Strengthening the frontline*’ states ‘*Strong primary health care – organized in multi-disciplinary teams and with innovative roles for health professionals, integrated with community health services, equipped with digital technology, and working with well-designed incentives – helps deliver a successful health system response. The innovations introduced in response to the pandemic need to be maintained to make health systems more resilient against future public health emergencies, and able to meet the challenges of ageing societies and the growing burden of chronic conditions’*. (OECD, 2021 p.1). The Expert Panel on Effective Ways of Investing in Health, advising the European Commission, published an Opinion on ‘*The Organization of resilient health and social care following the COVID-19 pandemic*’ recommending that ‘*Strong primary care and mental health systems should form the foundation of any emergency and/or preparedness response. All Member States should re-assess their investments in primary care and mental health and strengthen the integration of these systems with public health at population level. Aggregated levels of psychological distress should be recognized as a public health priority that requires a rapid adoption of clear behavioral strategies to reduce the burden of disease and the mental health consequences of an unexpected event’*. (Expert Panel on Effective Ways of Investing in Health, 2020).

Strengthening PHC requires actions at different levels: governance, patient flows (gate-keeping role), education and training, recruitment and retention, trust of the population in PHC and in the providers and, most importantly, financing. According to OECD Health Statistics 2018, 22 OECD countries spent on the average 13.6 % of their total health expenditures (THEs) on primary care services, ranging from 18.3 % for Australia to 9.5 % for Switzerland. We think that, if PHC is expected to fulfill the roles described above in the different reports, 25 to 30 % of THE should go to primary care services in the near future. This will enhance sustainable access to quality health and social care services, provided by interprofessional teams (family physicians, nurses, social workers, pharmacists, community health workers, etc.) and supported by informal care givers, focusing on the ‘life goals’ of individuals. A simultaneous investment in prevention and public health services of up to 10 % of THE will enable the integration of primary care and public health that is so much needed to increase resilience and preparedness for new challenges. In this integrated approach, public health interventions can build on trust developed by person-centered primary care services.

Where can the money for PHC come from? There are four ways to increase the resources for PHC:

1. Increase the THE: during the COVID-19 pandemic, citizens of most countries have put health care at the ‘number 1’ position for their political priorities. Hence, an investment of 8 to 12 % of GDP seems necessary to respond to these aspirations.
2. As documented earlier, 30 % of THE should be invested in PHC.
3. There should be an international mechanism to pay for ‘transition costs’ when countries decide to shift from a hospital-centric toward a PHC-based health system. Such a country should first invest in strengthening primary care services before it can start, for example, decreasing its number of acute hospital beds. So, for 3–4 years, there will be a need for ‘double financing’, keeping the hospital capacity in place, while investing in PHC. It is worthwhile to explore how international agencies like the European Commission, World Health Organization, and World Bank could contribute to the financing of those ‘transition costs’.
4. In a recent ‘Perspective’ in the WHO Bulletin, we described an opportunity to increase financing of primary care in the context of the 30by2030 campaign (www.30by2030.net). When major donors launch a call for disease-oriented projects focusing on specific health conditions such as HIV, diabetes, mental health conditions, tuberculosis, malaria, or coronavirus disease (COVID-19) in a country: ‘*applicants should make clear how they are going to improve primary health-care service delivery and channel 30% of the resources into strengthening PHC services. This strengthening can be done by financing the cost of integrating the project in the local primary health-care system. Achieving such integration requires contributing to capacity building for primary health care, supporting infrastructure upgrading, strengthening leadership and organization and improving the community’s involvement. However, characteristics of high-quality care such as accessibility, continuity, coordination and person-centeredness are also needed’*. (De Maeseneer et al., 2020). This mechanism creates a win-win for the population and for the health system. For more information, visit the website and sign the petition to support the campaign.

There is now a ‘window of opportunity’ to strengthen PHC and put the Alma Ata and Astana Declarations into practice. We urgently have to seize this opportunity, because people need PHC nowadays more than ever!
